# Are Currently Available Joint-Specific Patient-Reported Outcome Measures Fit for Purpose to Assess the Outcome of Knee Arthroplasty?

**DOI:** 10.3390/jcm14228073

**Published:** 2025-11-14

**Authors:** Cailin J. de Wet, Nicholas D. Clement, Thomas R. Williamson

**Affiliations:** 1 Edinburgh Orthopaedics, Royal Infirmary of Edinburgh, Edinburgh EH16 4SA, UK; 2 Centre for Population Health Sciences, Usher Institute, Usher Building, The University of Edinburgh, 5-7 Little France Road, Edinburgh BioQuarter, Gate 3, Edinburgh EH16 4UX, UK

**Keywords:** knee arthroplasty, PROMs, patient-reported outcome measures

## Abstract

Patient-reported outcome measures (PROMs) are integral to assessing patient function, quality of life, and pain both before and after knee arthroplasty (KA). Given the frequently excellent outcomes associated with KA, meaningful comparisons between patient cohorts or interventions require validated, precise PROMs that reliably reflect patient priorities and satisfaction. However, patients undergoing KA today have different demographic characteristics, different expectations, and different outcomes from those for whom these metrics were originally designed. Given changing lifestyles, many items in legacy PROMs may no longer represent priorities for patients in their postoperative recovery, and current evidence suggests that many are not associated with patient satisfaction. The frequent ceiling effects observed in PROMs for some subgroups following KA limit their reliability and utility in assessing outcomes for high-functioning patients. Whilst combining multiple PROM tools can provide a better, more holistic overview of patient outcome, it carries with it a significant burden and feasibility restriction. Item response theory and computerised adaptive testing present opportunities to collect PROMs from patients in a convenient manner and minimise question burden. Contemporary PROM collection requires both these innovative collection tools and analytic techniques, and questions that reliably reflect the priorities of the modern-day patient undergoing KA.

## 1. Introduction

Patient-reported outcome measures (PROMs) have become an essential tool for evaluating the outcome following knee arthroplasty (KA) [1]. Before widespread PROM utilisation, KA outcomes have been typically assessed through objective clinician-reported assessments, including radiographic parameters, revision and reoperation rates, and standardised function tests such as sit-to-stand time, the stair climb test, and the six-minute walk test [2,3]. Whilst these metrics provide some standardisation in outcome assessment and allow comparison between interventions and patient groups, they typically overlook elements of patients’ postoperative outcomes that are central to satisfaction, such as pain, quality of life, and ability to perform specific functional tasks and daily activities [4]. Given the frequently excellent postoperative outcomes associated with contemporary KA, solely considering imprecise outcomes such as survivorship risks a significant type two error when assessing new interventions or techniques.

This concern has led to the development and validation of a range of PROMs tailored to the KA population [5]. These measurement tools allow the assessment of a range of outcomes following KA, including those most pertinent to patients [4]. This resultant focus on patient-oriented outcomes has also allowed PROMs to be used in a wide range of clinical and academic settings, such as evaluating intervention cost-effectiveness [6].

These tools also enable PROMs to be utilised more broadly; for example, in registry-based outcome monitoring, health-economic evaluation, value-based care models, and comparative effectiveness research. PROM adoption in arthroplasty services has been considerable, with many national joint registries, healthcare systems and research consortia now mandating or encouraging routine PROM collection. In the UK, NHS England has employed routine collection for both hip and KA since 2009, and a collaborative national PROMs network was created in 2019 [7,8]. Despite incentives to improve PROM completion, substantial heterogeneity persists in PROM implementation [9], including variability in timing, completion rates, and selection of instruments, which complicates comparison across studies and health systems. This inability to carry out direct comparison significantly limits outcome interpretation. Many PROMs also lack appropriate validation and assessment of their measurement properties in this population [5,10].

Moreover, outcomes following KA can be confounded, and assessment of PROMs reflects multifactorial, biopsychosocial constructs rather than simple markers of implant success. Interpretation needs to account for alignment phenotype, preoperative psychological state, surgical technique, and instrument validity within the studied population [11,12]. This is complicated further by the established interactions between patient demographics, knee pathology, psychological factors, and native anatomy and alignment, which all can impact outcomes postoperatively [13]. A deeper understanding of these interactions is essential for advancing personalised alignment strategies, improving outcome interpretation, and refining the use of PROMs as valid indicators of patient-centred recovery after KA.

## 2. Old Tools and Modern Patients

PROM utilisation in KA is increasing year-on-year, with the number of studies reporting PROMs following surgery nearly doubling between 2010 and 2020 [14]. This reflects a move towards a more multidimensional, patient-centred approach to outcome assessment. PROMs assessing joint-specific function, such as the Oxford Knee Score (OKS), Knee Injury and Osteoarthritis Outcome Score (KOOS), and Forgotten Joint Score (FJS), have become well-established following KA for their ability to capture pain, stiffness, functionality, and health-related quality of life from the patient’s perspective [15,16,17].

There is a large body of evidence that supports these as validated outcome measures following KA [5]. However, many of these metrics were developed in the last century, and for different patient populations than the modern-day patient following KA. For example, the original descriptions of the OKS and KOOS were first published in the 1990s, with the OKS designed alongside 20 patients awaiting KA, and the KOOS with a cohort of patients post-meniscectomy [15,16]. This has led to debate surrounding their long-term clinical value, with evidence suggesting diminishing benefit reflected in the PROMs as the length of follow-up increases [18].

Dawson et al. originally reported item-level OKSs for patients undergoing KA in 1998 [15]. A recent study found the overall OKS was 5 and 6 points greater pre- and postoperatively [19], respectively, when compared with the original population described by Dawson et al. [15]. The reason(s) for this are likely to be multifactorial and may reflect both changing patient expectations and a loss of relevance of some individual questions, given the change in lifestyle since the score was designed [20]. A large multicentre audit conducted in Germany identified that patients’ expectations ahead of KA were influenced by their demographics, including age, sex and body mass index (BMI) [21]. Expectations of KA are also independently associated with individual patients’ postoperative outcomes as assessed by PROMs [22,23,24]. This interplay of patients’ demographics, expectations, and outcomes is important, given that the frequency of obesity in patients awaiting contemporary KA is higher than when these tools were developed [25], and patients are often undergoing KA at a younger age [26], which are both factors associated with differing expectations and outcomes following KA [27,28].

Patients’ expectations regarding KA not only relate to their anticipated postoperative improvement in symptom burden, but also to their inpatient experience and patient journey [22,29]. In recent years, there has been a growing interest in the development of novel patient pathways for patients undergoing KA, focusing on enhanced recovery after surgery [30]. ‘Same-day’ KA has significantly grown in popularity, increasing by over 10% per annum in recent years [31]. Whilst the careful selection of patients suitable for ‘same day’ KA is imperative, appropriately selected patients have been shown to have high rates of satisfaction and willingness to re-undergo same day surgery should they need another future KA [32]. Meanwhile, commonly used knee-specific PROMs, such as the OKS or KOOS, assess overall knee health and do not specifically capture patellofemoral symptoms. Conversely, patellofemoral-focused PROMs, such as the Kujala Anterior Knee Pain Scale, evaluate anterior knee function but not global knee performance. This highlights a gap in current outcome assessment, suggesting that combined or more comprehensive instruments may better capture both patellofemoral and tibiofemoral function [33].

In addition, further complexity in assessing patient outcome lies in that heterogeneity in operative technique has a significant impact on post-operative outcomes, but many elements of this remain poorly understood. Difficulty in controlling for the significant number of interacting factors dramatically limits the ability of PROMs to discriminate between KA techniques. Differing alignment philosophies often result in differing flexion and extension gap characteristics, for example, mild asymmetry in flexion may be tolerated in functionally aligned KA, but not in mechanical alignment [34,35]. A previous lack of standardisation in alignment strategy terminology and calculation has further complicated outcome assessment further [34], and whilst considerable attempts have been made to standardise both alignment strategy classification and coronal plane angle calculations, it remains unclear what impact this has on outcomes [36]. Handling of the patellofemoral compartment, including both patella resurfacing and the avoidance of over- or understuffing of the trochlea in KA, may impact outcomes postoperatively [37,38]. The emergence of robotic-assistance has complicated this further, as various systems with differing haptic boundaries are employed worldwide, whilst still not controlling for the heterogeneity in operative technique, implant choice, and alignment strategy utilised by the surgeon [39]. Recent evidence from the FP-UCBM Knee Study Group demonstrated that unrestricted kinematic alignment in varus KA yielded superior PROMs compared with mechanical alignment in patients with a Coronal Plane Alignment of the Knee (CPAK) classification I phenotype, but comparable outcomes in CPAK IV [12].

Factors impacting outcomes from KA are not limited to biomechanics, as a prospective comparative study showed that preoperative anxiety and depression were independently associated with worse postoperative pain and subjective function following KA, despite demonstrating no relevant clinical differences [11]. Furthermore, it is important to consider the interaction between gender, knee anatomy and native alignment, and KA outcomes. Female patients typically experience significantly worse preoperative PROMs than male patients whilst awaiting KA [33]. Patient gender has been shown to impact native alignment, with neutral and valgus phenotypes more frequently expressed in female patients, whilst male patients express predominantly varus phenotypes [13]. This results in differences in surgical technique and intraoperative soft tissue handling, as the necessity for soft tissue releases to achieve an adequately balanced KA is directly impacted by the pattern of OA and extent of deformity [34]. Nonetheless, despite this interaction between gender and intraoperative technique, studies also demonstrate no significant differences in post-operative PROMs between genders [33,35,36]. Similarly, socioeconomic status is strongly linked to outcomes following arthroplasty generally, with studies demonstrating links between income, education, employment, and PROMs [40]. Other evidence highlights that factors more prevalent in lower socioeconomic groups were significantly correlated with reduced improvement in their OKS and overall dissatisfaction post-KA [41]. In contrast to this finding, another paper has shown comparable PROMs across social groups [42].

Collectively, these findings highlight that both biomechanical and psychosocial determinants substantially influence patient-reported outcomes, and the widespread interactions between these elements significantly limit PROMs’ ability to determine the true effect of a change in KA technique or patient care. Moreover, patients’ expectations for their KA in the setting of changing patient demographics and surgical pathways are dynamic. To employ legacy PROMs, although validated, to assess the outcome of patients over a quarter of a century after they were designed could be questioned.

## 3. Clinimetric Properties, Interpretability and Feasibility

The OKS is widely used to assess joint-specific symptoms before and after KA, with an average 15-point improvement observed at 12 months [19]. A 7-point improvement in the OKS is considered clinically meaningful [43]. However, the interpretation of meaningful values for legacy PROMs, such as the ‘minimal clinically important difference’ (MCID) and the ‘patient acceptable symptom state’ (PASS), has been limited by the heterogeneity in assessment and calculation methods [44]. A wide range of calculation techniques exist, with variations in both ‘distribution-based’ methods and ‘anchor-based’ methods described [45]. ‘Distribution-based’ methods typically rely on determining meaningful values from the collected data using statistical reasoning alone, whilst ‘anchor-based’ methods rely on the use of an anchor question, frequently related to satisfaction, to contextualise the PROM scores and allow the calculation of a corresponding MCID or PASS [46]. Franceschini et al. highlighted that using differing calculation techniques resulted in a wide variation in the defined MCID values for the International Knee Documentation Committee (IKDC), despite using a standardised anchor question for their anchor-based methodologies [47]. Whilst variations in calculation methodologies may result in profoundly differing meaningful values reported, further concerns lie in that the specific anchor questions used for ‘anchor-based’ methods vary widely, with no standardisation in either question content or timing across studies or populations [44]. The impact of this methodological heterogeneity cannot be reliably quantified when comparing studies. It remains unclear whether differences in the meaningful values reported for legacy PROMs across different populations relate to intrinsic differences between the populations compared or are simply due to the methods employed.

Furthermore, how the change in OKS relates to individual symptoms remains unclear. It has been shown that item-level score improvement varies from 0.7 (washing) to 2.2 (limping) and is influenced by baseline symptom severity and patient demographics, with greater improvements seen in those with worse preoperative scores [19]. Discrete latent classes have been identified in OKS recovery, with distinct subgroups of function and pain assessed, although both outcomes are frequently interdependent in the KA population [48,49]. Taking the overall change in OKS or postoperative score in isolation may overlook distinct patterns of recovery for patients and makes recovery in one domain directly comparable with another, despite this not necessarily reflecting patients’ priorities. Indeed, differences in association between individual OKSs and satisfaction have been shown for patients undergoing KA, with pain scores more likely to be associated than function [19]. Given that not all 12 questions appear equally relevant, future PROM design should consider which symptoms and outcomes are priorities for contemporary KA patients.

A further limitation with using the OKS to assess outcome following KA is that the literature has shown mixed evidence of ceiling effects, with up to 33% of patients reaching the top score following KA [50]. While some studies found no ceiling effect, subgroup analyses suggest that male and older patients are more likely to achieve the highest scores, potentially reflecting differences in expectations and symptom prioritisation [51]. The structure and content of PROMs may contribute to ceiling effects, and scales with fewer items, such as the OKS, are more prone to clustering at the top end of the scale [10]. In contrast, tools like the FJS are designed to reduce ceiling effects by tailoring questions to individual activity levels [52]. However, even these tools can disadvantage certain groups; for instance, elderly patients may struggle to answer questions about sports they may no longer engage in.

Combining joint-specific PROMs with generic quality-of-life measures, such as the EQ-5D, may help capture broader health outcomes [53]. However, this significantly increases the response burden for patients, especially in older adults. Response burden is a core principle of PROM feasibility and is integral to instrument selection [54]. Increasing questionnaire burden has been shown to directly impact response rates, alongside age and other demographic parameters, with increased burden inversely associated with response frequency [55]. Loss to PROMs follow-up is of such a particular concern following KA that concepts such as the clinical relevance ratio (CRR) have been introduced to attempt to mitigate against the associated bias, aiming to make results more representative and meaningful [56]. Loss to follow-up must be minimised to ensure that subgroups of patients less likely to respond, such as younger patients and those with more severe disability and functional impairment, are not disproportionately disadvantaged [57].

Given that patients often undergo longitudinal follow-up and multiple assessments following KA over many years, it would seem beneficial to shift the focus from quantity to content, ensuring that PROMs reflect what matters most to patients today. As arthroplasty outcomes improve and patient expectations evolve, PROMs must adapt to remain meaningful and relevant. However, while efforts to minimise patient burden and rationalise the number of questionnaire items are important, these must be balanced against the need to capture sufficiently comprehensive data, as no single instrument can adequately represent all patient groups.

## 4. Current Trends and Technological Advances

Whilst paper-based questionnaire-style methods of collecting PROMs are well established, there are also many newer developments in the field. One of these is the item response theory (IRT), which is a mathematical method of quantifying the relationship between a patient’s response and ‘latent characteristics’, or position on the continuum of what is being measured [49]. This concept can be combined with computerised adaptive testing (CAT) to dynamically administer the items (questions) that provide the most information given an individual’s previous responses [58]. CAT has been used to apply IRT to the national PROMs database and has been demonstrated to achieve the same precision rate with fewer questions, thus reducing patient burden while retaining high measurement precision across the spectrum of health states [49]. Given that one of the main challenges in using PROMs to assess KA outcomes is the difficulty in obtaining adequate response rates, optimising PROM feasibility may enable enhanced data gathering and more meaningful interpretation [57]. The advent of IRT and CAT represents a promising avenue to improve patient experience in PROM administration and increase understanding of outcomes following KA [59]. However, the content of the questionnaire administered relies on traditional PROM items, such as those from the OKS and KOOS, and whether these are relevant and related to patients’ satisfaction following KA is not clear [60].

The use of smartphones and patient portals has facilitated PROM collection, offering a convenient, cost-effective way to gather data directly from the patient, both preoperatively and at multiple points after surgery [61]. There are improved completion rates when surveys are administered via phone applications [61], and there is evidence that a multimodal approach may further increase the ability to capture PROMs [62]. Moreover, the patients are satisfied with this method of survey distribution, which supports the idea that using smartphone technology reduces the patient burden when it comes to collecting this data [63]. These studies highlight how utilising existing technology can potentially increase engagement with PROM monitoring and potentially simplify the process in a way that is acceptable to patients.

As well as smartphone technology, wearable technologies are an adjunct that may allow the collection of additional objective data [64,65]. Many patients wear items such as smartwatches with inbuilt pedometers or use their phones to track step counts and other data. It has been demonstrated that these can effectively characterise patients’ activities in more detail quantitatively, allowing better individualisation of therapy goals [66]. Huffman et al. [67] observed that step count, as measured by wearable technology, was positively correlated with PROMs following total hip arthroplasty. Combining objective data with the responses to PROMs may strengthen the conclusions researchers can draw, leading to more robust evidence-based improvements in care. Although barriers to the adoption of wearable technology exist among older adults, the decreasing age of patients undergoing knee arthroplasty [22] and the emergence of a more technologically literate generation are likely to enhance the feasibility and impact of such data collection methods.

The routine collection of PROMs, supported by digital health platforms, is increasingly integrated into clinical practice and research. Orthopaedics is a frontrunner in the field as one of the early adopters to start amassing population-level PROMs data for hip and knee arthroplasty, and national registries now routinely incorporate PROMs alongside traditional outcomes [68]. This allows for population-level analyses of satisfaction, functionality, and health-related quality of life [69]. These ‘big data’ population datasets allow the granular analysis of outcomes associated with specific cohorts of patients and specific implants, providing meaningful insights for clinicians and patients [68].

In addition to advances in technology for the collection and interpretation of PROMs, robotic-assisted KA represents a recent enormous development in the field. The use of robotic-assisted surgery is now widely accepted within orthopaedics [70]. Some studies report that it also confers improved PROMs compared to conventional KA, although others find that these differences are not statistically or clinically significant [71,72].

## 5. Future Directions

Building on the established precedent for embracing technological advances within orthopaedics and specifically KA, it presents an ideal field for further incorporating technology in pre-, peri- and postoperative care. In order to fully utilise the recent advancements, tools like CAT and IRT need to be made more widely available. This would allow researchers and clinicians to deliver PROMs most efficiently, reducing patient burden and increasing data capture. The creation of more personalised outcome metrics may further improve patient engagement and response rates, as PROMs may seem more directly relevant to the patient’s goals and expectations.

With an ageing population, patients are living longer following joint arthroplasty [73]. This may result in extended follow-up and repeated PROM administration over longer periods. Combining PROM recovery trajectory with revision and reoperation data may allow the detection of patients at risk of early failure and revision arthroplasty and enable closer monitoring or more targeted follow-up. However, long-term PROM interpretation requires caution, as it is currently unclear whether deterioration in long-term PROM scores truly reflects declines in joint function or is merely a product of age-related changes in quality of life and global function.

Artificial intelligence (AI) techniques are developing rapidly, leading to increased widespread adoption. The use of machine learning (ML), a subset of AI, to reliably predict and classify novel data from ‘training’ datasets is being increasingly utilised within orthopaedics and arthroplasty [74]. These techniques have the potential to revolutionise care for patients following KA and significantly enhance the utility of PROMs in patient care. The potential benefits are widespread, including in predicting long-term outcomes from patient-specific, surgery-specific and radiographic parameters; using natural language processing (NLP) to capture broad and personalised insights regarding patient outcomes; and through optimising techniques such as IRT and CAT. Prediction of long-term outcomes from patient characteristics, surgical parameters, and early functional data could dramatically reduce the long-term burden of PROM collection, allowing targeted and personalised PROM follow-up of the subset of patients at risk of poor recovery [75]. This cohort of ‘at risk’ patients may require adapted PROM tools or personalised assessment, as traditional PROMs were not designed to assess such a patient population. Furthermore, ML techniques may be used to improve traditional IRT and CAT techniques, progressing from traditional Bayesian techniques to personalised classification [76]. Recent research has aimed to evaluate the use of unsupervised IRT models in optimising differential item functioning and accommodating for latent classes within response groups [77], although recent findings in patients undergoing cardiac angiography report inconsistent outcomes in commonly used models, highlighting the need for further development [78].

An alternative mechanism through which AI could aid PROM utility is through the combination of free-text responses in outcome assessment, such as via a large language model (LLM) and NLP. Existing LLMs are sensitive but lack specificity in determining functional outcomes from patients’ notes alone, with further work proposed to improve model performance [79]. The combination of patients’ free text answers and NLP could enable the codification and categorisation of patients’ responses, allowing subsequent objective outcome assessment. Such outcomes would be vastly different from current PROMs and would require rigorous interrogation and validation, but could provide an even more patient-centred approach to outcome assessment. However, the introduction of AI systems to aid PROM utility and patient outcome assessment is not without its risks. Such developments would require careful planning to mitigate against these systems’ dependence on digital infrastructure and global networks, and to ensure that treatment decision making remains at the discretion of a trained clinician, not an algorithm. The application of AI to PROM analysis should complement, not supplant, the interpretive role of the clinician; integration of such tools must remain anchored in clinical reasoning and the therapeutic relationship.

Overall, there is growing recognition that a “one-size-fits-all” approach to outcome assessment may not adequately reflect the diversity of patient expectations and experiences, nor the demands of the modern patient. Whilst this will need careful design to ensure standardisation and allow comparison between patient cohorts and interventions, tailoring instruments to patients’ current priorities and expectations could enhance their utility in guiding care and assessing outcomes.

## 6. Conclusions

Current developments in PROMs after KA reflect a move toward patient-centric, precise, and adaptable measures that can be integrated into routine care at scale. The future lies in personalised, technology-assisted instruments that help surgeons, healthcare providers, and health systems better appreciate and respond to the patient’s view of their health and recovery, whilst still allowing a degree of standardisation that allows comparison of outcomes on a population scale.

## Data Availability

Not applicable.

## Abbreviations

The following abbreviations are used in this manuscript:

PROMs	Patient-reported outcome measures
KA	Knee arthroplasty
OKS	Oxford Knee Score
KOOS	Knee Injury and Osteoarthritis Outcome Score
FJS	Forgotten Joint Score
BMI	Body mass index
CPAK	Coronal Plane Alignment of the Knee
MCID	Minimal clinically important difference
PASS	Patient acceptable symptom state
IKDC	International Knee Documentation Committee
CRR	Clinical relevance ratio
IRT	Item response theory
CAT	Computerised adaptive testing
AI	Artificial intelligence
ML	Machine learning
NLP	Natural language processing
LLM	Large language model
